# Exploring the Potential of Precision Medicine in Neuropsychiatry: A Commentary on New Insights for Tailored Treatments Based on Genetic, Environmental, and Lifestyle Factors

**DOI:** 10.3390/genes16040371

**Published:** 2025-03-24

**Authors:** Jelena Milic, Milica Vucurovic, Dragana Jovic, Veroslava Stankovic, Edita Grego, Srdja Jankovic, Rosa Sapic

**Affiliations:** 1 Institute of Public Health of Serbia “Dr Milan Jovanović Batut”, 11000 Belgrade, Serbia; 2 European Faculty “Kallos”, Ratariski Put 8a, 11000 Belgrade, Serbia; 3 The College of Health Science, Academy of Applied Studies, 11000 Belgrade, Serbia; 4 The University Children’s Hospital, 11000 Belgrade, Serbia; 5 Faculty of Health Studies, University of Bjeljina, 76300 Bjeljina, Bosnia and Herzegovina

**Keywords:** precision medicine, neuropsychiatric disorders, genetics, pharmacogenomics, gene–environment interactions, schizophrenia, bipolar disorder, personalized treatment

## Abstract

Neuropsychiatric disorders are complex conditions with multifactorial etiologies, in which genetics play a pivotal role. Despite significant advancements in psychiatric research, traditional treatment options remain largely symptomatic, focusing on clinical signs without fully addressing the underlying biological causes. However, recent developments in precision medicine—an approach that tailors treatments based on genetic, environmental, and lifestyle factors—hold great promise for transforming the treatment of these disorders. By identifying specific genetic markers and understanding gene–environment interactions, precision medicine can offer more personalized and effective treatments, leading to better patient outcomes. Our primary aim was to explore how integrating genetic data with environmental factors could enhance the understanding and treatment of neuropsychiatric conditions such as schizophrenia, bipolar disorder, and depression. The secondary aim was to examine the potential of pharmacogenomics and gene therapy in improving therapeutic strategies. The results indicate that while significant progress has been made, challenges remain, including the complexity of genetic interactions and the need for more granular phenotypic data. In conclusion, precision medicine has the potential to revolutionize neuropsychiatric treatment by providing individualized care that considers genetic makeup, environmental influences, and lifestyle factors, paving the way for more effective therapies and improved patient outcomes.

## 1. Introduction

### 1.1. Purpose and Context

Neuropsychiatric disorders are complex conditions that represent a significant global health burden with complex and multifactorial etiologies [1]. Despite significant advances in psychiatric research, traditional treatment options for these disorders remain largely symptomatic, focusing on deviating clinical signs without fully addressing the underlying biological causes. However, recent advancements in precision medicine—an approach that tailors treatment based on an individual’s genetic, environmental, and lifestyle factors—hold the promise of transforming the treatment of neuropsychiatric disorders. By identifying specific genetic factors and understanding the interplay of environmental influences, precision medicine can lead to more personalized effective treatments and offer a more nuanced understanding of the pathophysiology of these complex conditions [2]. A growing body of work has produced compelling evidence linking genetic factors and environmental interactions to a wide range of human diseases [3]. This commentary explores the potential of precision medicine to revolutionize neuropsychiatry, emphasizing its role in uncovering the biological mechanisms behind psychiatric disorders and improving treatment outcomes.

### 1.2. Epigenetic Modifications in Neurodegenerative Diseases: Insights from Recent Research

Recent research has particularly focused on the role of DNA methylation (a process where chemical tags are added to DNA, altering gene expression without changing the DNA sequence) and histone modifications (changes to proteins around which DNA is wrapped, affecting how tightly or loosely the DNA is packaged, which influences gene activity) in both brain tissue and blood, emphasizing their significant role in influencing disease development and progression in neuropsychiatric disorders, including Alzheimer’s and Parkinson’s diseases [4]. Studies examining the methylation of specific genes, such as MAPT, reveal that methylation patterns can vary by tissue type, highlighting tissue-specific epigenetic changes in these diseases [5]. In Alzheimer’s disease (AD), the methylation of genes like BDNF has been linked to neurodegenerative processes, with hypomethylation and hypermethylation patterns observed in different brain regions [4]. Similarly, Parkinson’s disease (PD) shows differentially methylated genes such as SNCA, which are consistently hypomethylated in both peripheral blood and brain tissue, suggesting a potential biomarker for PD progression [4]. Genome-wide approaches have further identified differential methylation patterns in both blood and brain tissue, reinforcing the significance of these modifications across different tissues and providing insight into the epigenetic regulation of neurodegenerative diseases [4].

Importantly, these findings emphasize that the methylation of genes, such as MAPT in PD, differs across tissues, suggesting that both brain and peripheral tissue profiles can offer valuable insights into disease mechanisms and potential diagnostic markers [4]. The global representation of these studies, which included participants from diverse regions such as the USA, China, Canada, and Europe, highlights the widespread interest in understanding these genetic and epigenetic factors across different populations, further contributing to the global effort in combating neurodegenerative diseases [4].

In the context of precision medicine, understanding these epigenetic modifications not only enhances our knowledge of disease pathogenesis but also paves the way for more personalized therapeutic approaches [6]. By identifying specific epigenetic alterations as biomarkers, clinicians can potentially use this information to tailor treatments to the unique genetic and epigenetic profiles of patients [7]. For instance, targeted therapies could be designed to reverse or modify detrimental methylation patterns or histone modifications that are contributing to disease progression, ensuring that treatments are more effective and individualized. Moreover, the integration of genetic and epigenetic data from both brain and peripheral tissues can provide a more comprehensive understanding of the disease, guiding early diagnosis and monitoring treatment responses [8]. This personalized approach, driven by a deeper understanding of epigenetic regulation, has the potential to revolutionize the treatment of neurodegenerative diseases, much like in other fields of precision medicine, and could lead to better outcomes for patients suffering from these debilitating conditions.

#### 1.2.1. Addressing the Need for Precision Medicine in Neuropsychiatric Disorders

While the genetic and epigenetic landscape of neuropsychiatric disorders such as depression has been extensively studied, it is crucial to highlight the limitations of current treatment approaches. Traditional pharmacological treatments for depression, including selective serotonin reuptake inhibitors (SSRIs) and tricyclic antidepressants, are often prescribed based on symptoms alone, without considering the genetic and epigenetic factors that could influence treatment response [9]. This approach leads to a trial-and-error process, where patients may experience delays in finding an effective treatment or may undergo adverse side effects. Recent studies suggest that genetic variations, such as those in serotonin transporter genes, and epigenetic modifications, including DNA methylation patterns, can significantly affect an individual’s response to antidepressants [10]. For example, patients with certain genetic polymorphisms in the serotonin transporter gene (SLC6A4) may respond better to SSRIs, while others may not experience significant benefits. Furthermore, epigenetic changes influenced by early-life stress or chronic environmental factors could alter the expression of genes involved in mood regulation, further complicating treatment outcomes. By incorporating genetic and epigenetic profiles into clinical practice, physicians can better predict treatment responses, avoid ineffective therapies, and select more targeted interventions that could result in faster recovery and fewer side effects [11]. This approach represents a critical shift toward precision medicine in psychiatry, ensuring that treatments are tailored to the unique genetic makeup of each patient, ultimately improving both efficacy and patient outcomes.

#### 1.2.2. The Role of Precision Medicine in Neuropsychiatric Disorders

Precision medicine is transforming the way we approach neuropsychiatric disorders, providing new opportunities for personalized treatment strategies. By integrating genetic, epigenetic, and environmental data, precision medicine aims to tailor interventions that are more effective and better suited to the unique needs of each patient [12]. Neuropsychiatric disorders, including depression, schizophrenia, and bipolar disorder, are complex and multifactorial, with a strong genetic component. However, the interplay between genetics, environmental factors, and lifestyle choices also plays a crucial role in disease onset and progression [13].

Recent advances in genomic and epigenomic research have revealed specific genetic variations and epigenetic modifications that influence individual susceptibility to these disorders and their response to treatment. For instance, variations in genes related to neurotransmitter regulation, such as the serotonin transporter gene (SLC6A4), have been associated with different responses to antidepressants. Similarly, epigenetic modifications like DNA methylation can affect gene expression involved in mood regulation and stress responses, influencing how patients with depression or other psychiatric conditions respond to therapeutic interventions [14].

The incorporation of these findings into clinical practice allows for more individualized treatments, moving beyond the trial-and-error approach traditionally used in psychiatry. By understanding the genetic and epigenetic profiles of patients, healthcare providers can better predict treatment outcomes, minimize adverse effects, and select the most appropriate interventions.

Precision medicine’s role in neuropsychiatric disorders is particularly significant in the context of depression, which is one of the most prevalent psychiatric conditions globally [15]. With advances in precision medicine, we can now personalize therapeutic strategies, providing patients with treatments that are tailored to their specific genetic makeup, lifestyle, and environmental exposures. This shift promises not only to improve treatment efficacy but also to enhance our understanding of the pathophysiology of neuropsychiatric disorders, ultimately leading to better outcomes and improved patient care.

### 1.3. The Role of Genetic Markers in Neuropsychiatric Disorders

There is a growing emphasis on the role of genetic factors (inherited traits and variations in genes that contribute to an individual’s risk of developing specific conditions) in the neuropsychiatric disease field. Recent studies have highlighted the relationship between polygenic risk scores and the individual risk for major depression, as well as the significant genetic overlap between depression and other psychiatric disorders [16]. Delta Electrocardiographic Depression (δEPCD), a novel physiological biological marker (measurable indicators of biological processes or conditions that can help diagnose or monitor a disease), shows potential but remains influenced by both static and dynamic biological factors [17]. This complexity makes it a promising yet not entirely reliable tool in clinical settings [18]. Such findings highlight the need to increasingly focus on genetic factors and personalized medicine approaches, as they offer more consistent and clinically actionable insights [18]. As precision medicine continues to evolve, these genetic factors may become integral in more accurately diagnosing and treating depression, shifting from symptom-based treatments toward more individualized targeted interventions.

#### Clarifying the Role of Genetic and Epigenetic Markers in Neuropsychiatric and Neurodegenerative Disorders

The distinction between genetic and epigenetic markers is crucial when exploring the pathophysiology of neurodegenerative and neuropsychiatric disorders [19]. While both types of markers are involved in disease development, their roles and mechanisms differ significantly, particularly when examining the interplay between genetics, the environment, and disease expression.

Genetic Markers in Neuropsychiatric Disorders

Genetic markers are widely studied in neuropsychiatric disorders, particularly in conditions such as depression, schizophrenia, and bipolar disorder [20]. These disorders are often linked to specific genetic variations, where changes in the genetic code or mutations in particular genes can influence an individual’s susceptibility to psychiatric conditions [21]. In depression, for example, specific genes related to neurotransmitter regulation—such as those involved in serotonin and dopamine pathways—are believed to play a key role in the development of symptoms [22]. Genetic studies, including genome-wide association studies (GWAS), have identified multiple loci associated with these disorders, which help predict susceptibility, disease onset, and response to treatment. Genetic markers in neuropsychiatric disorders are typically associated with direct alterations in brain function, often related to neurotransmitter systems or neural signaling pathways [23].

Epigenetic Markers in Neurodegenerative Disorders

In contrast, epigenetic markers are more frequently discussed in the context of neurodegenerative diseases such as Alzheimer’s disease, Parkinson’s disease, and Huntington’s disease [24]. Epigenetic changes do not involve alterations in the DNA sequence but rather modifications that influence gene expression. These modifications are influenced by environmental factors such as diet, stress, toxins, and infections, which can lead to long-lasting changes in gene activity [25]. Epigenetic changes, such as DNA methylation, histone modification, and non-coding RNA expression, can impact the development and progression of neurodegenerative disorders [26]. For instance, in Alzheimer’s disease, epigenetic modifications are thought to affect genes involved in amyloid precursor protein processing, which plays a central role in disease progression. Environmental influences, such as exposure to oxidative stress or inflammation, can exacerbate these epigenetic changes, leading to neuronal damage and cognitive decline.

Interrelationship Between Genetic and Epigenetic Factors

While genetic markers play a predominant role in neuropsychiatric disorders and epigenetic factors are more often highlighted in neurodegenerative diseases, there is growing evidence of the interplay between these two mechanisms. In both fields, the environment can modify genetic expression through epigenetic changes, blurring the line between genetic predisposition and environmental impact [27]. For example, in depression, early life stressors or trauma may lead to epigenetic changes in genes related to stress response and mood regulation. Similarly, in neurodegenerative diseases, genetic risk factors may interact with environmental exposures to accelerate disease progression through epigenetic modifications [28].

Understanding the roles of both genetic and epigenetic markers in these disorders is essential for developing more accurate diagnostic tools and personalized treatment strategies. The integration of genetic and epigenetic information will enable a more comprehensive understanding of disease mechanisms and improve precision medicine in psychiatry and neurology.

### 1.4. The Role of Genetic and Environmental Determinants in Mental Health and Public Health

The understanding of mental health disorders has traditionally focused on genetic predispositions, yet an increasingly nuanced view highlights the importance of environmental and social determinants. Genetic research has significantly advanced our knowledge of the molecular underpinnings of psychiatric conditions, but these conditions cannot be fully understood without considering the role of environmental and social factors [21]. Environmental factors, such as childhood trauma, stress, socioeconomic status, substance abuse, and access to mental health care, play a crucial role in either triggering or exacerbating psychiatric symptoms in individuals with a genetic predisposition. Similarly, social determinants like social support, community cohesion, and education are known to either buffer or intensify the effects of genetic risk factors [21].

Incorporating both genetic and environmental perspectives allows for a more holistic understanding of mental health, emphasizing that psychiatric disorders result from complex interactions between inherited vulnerabilities and environmental exposures [29]. The public health implications of this integrated approach are profound, as it calls for targeted prevention strategies and policies that address not only genetic risk factors but also the social and environmental contexts that shape mental health outcomes at the population level. For instance, addressing childhood adversity and improving access to mental health services could substantially reduce the incidence of psychiatric disorders in at-risk populations [30].

This dual focus on genetics and environment is critical for advancing precision psychiatry, which tailors treatment to the specific needs of individuals [31]. It also underscores the need for public health initiatives aimed at creating environments that foster mental well-being, providing early interventions, and promoting mental health education. By considering both genetic and environmental influences, we can better understand how to mitigate risk factors and promote mental health in diverse populations.

### 1.5. Key Question

The core issue explored in this commentary is whether precision medicine can effectively unlock the pathophysiology of neuropsychiatric disorders, such as bipolar disorder, schizophrenia, and depression, by integrating genetic, environmental, and molecular data for personalized treatments.

### 1.6. Scope

This commentary delves deeply into the transformative role of precision medicine in neuropsychiatry, with a particular focus on its potential to personalize treatment for neuropsychiatric disorders like depression, schizophrenia, and bipolar disorder. We examine how both genetic and environmental factors contribute to the onset and progression of these disorders, underscoring the value of precision medicine in providing personalized therapeutic approaches. In addition to genetic markers, we address epigenetic factors—such as DNA methylation and histone modifications—and how they further refine treatment strategies. Gene–environment interactions are discussed as essential elements in understanding the complex pathophysiology of these conditions, suggesting that future therapeutic efforts should not only target symptoms but also consider the unique genetic and environmental profiles of individuals. This precision-driven approach is poised to reshape the landscape of mental health treatment, improving efficacy and minimizing adverse side effects. Through the integration of both genetic and environmental data, clinicians can make more informed decisions, ultimately leading to better patient outcomes and contributing to the evolving field of precision psychiatry.

## 2. Discussion

### 2.1. Current Knowledge and Gaps

Recent breakthroughs in genetic research have identified various risk factors associated with psychiatric disorders [32]. Large-scale genome-wide association studies (GWAS) have pinpointed several loci linked to conditions such as bipolar disorder, schizophrenia, and depression, providing critical insights into their molecular underpinnings. Psychiatric genetics has advanced significantly, shedding light on the genetic causes of psychiatric disorders and enabling precision psychiatry [33]. This approach uses genetic profiles to personalize risk assessment and clinical decisions. While psychiatric disorders are known to be heritable, recent studies reveal that they result from thousands of genetic variants working together. These common variants contribute to genetic risk, with everyone carrying some level of susceptibility. Large-scale studies have identified both common and rare genetic variants linked to major psychiatric disorders [21]. For example, it has been known for over two decades that in bipolar disorder, variants in genes related to neurotransmitter systems, such as Catechol-O-methyltransferase, serotonin transporter, and tryptophan hydroxylase gene polymorphisms have been implicated [34]. Similarly, schizophrenia has been associated with mutations in genes governing synaptic function and neurodevelopment, such as the DISC1 and NRG1 genes [35].

Despite these advancements, the genetic contribution to psychiatric conditions remains poorly understood in its entirety. Many of the genetic variants identified through GWAS have small effect sizes and explain only a fraction of the heritability of these disorders [10]. To address this, precision medicine approaches aim to integrate genetic findings with other biological markers, including epigenetic modifications and biomarkers associated with brain function. For example, integrating gene expression data with neuroimaging studies could provide a more comprehensive picture of how specific genetic variants influence brain structure and function, offering new targets for therapeutic interventions.

Recent breakthroughs in genetic research have identified various risk factors associated with psychiatric disorders. Large-scale genome-wide association studies (GWAS) have pinpointed several loci linked to conditions such as bipolar disorder, schizophrenia, and depression, providing critical insights into their molecular underpinnings [36]. Psychiatric genetics has advanced significantly, shedding light on the genetic contributions to psychiatric disorders and enabling personalized psychiatry. However, while psychiatric disorders exhibit heritable components, it is important to emphasize that heritability varies across different disorders and does not guarantee that affected individuals will pass on the condition to their children [37]. The genetic predisposition to psychiatric conditions is complex and multifactorial, with many individuals who inherit certain risk factors not necessarily developing the disorder. Moreover, environmental factors, including childhood trauma, socio-economic stress, and access to mental health care, play a crucial role in the manifestation of psychiatric conditions [38]. Consequently, psychiatric disorders result from the interaction between genetic and environmental factors, and their heritability should be considered in this broader context. The identification of both common and rare genetic variants linked to major psychiatric disorders underscores the complexity of these conditions and emphasizes the need for an integrated approach that accounts for both genetic susceptibility and environmental influences [21].

#### Heritability in Psychiatric Disorders: Revisiting Genetic Contributions and Environmental Interactions

The heritability of psychiatric disorders is a well-established concept in genetic research, but it is essential to clarify that heritability does not imply deterministic outcomes. Psychiatric disorders, such as bipolar disorder, schizophrenia, and depression, show a strong genetic component, yet this genetic influence is far from absolute [39]. Genetic studies, including genome-wide association studies (GWAS), have identified multiple genetic variants linked to these conditions, but these variants often account for only a fraction of the heritability observed in populations. Importantly, heritability estimates for psychiatric disorders vary significantly across different studies, populations, and even within specific subtypes of disorders. For example, schizophrenia’s heritability has been estimated to be around 80%, but even in cases with a strong genetic component, not all individuals with genetic risk factors will develop the disorder [40]. This highlights the importance of understanding psychiatric disorders as the result of complex interactions between genetic and environmental factors.

While genetics plays a crucial role, environmental influences such as childhood trauma, stress, substance abuse, and socio-economic factors also significantly shape the development and progression of psychiatric disorders [41]. For instance, individuals with a genetic predisposition to depression may not manifest the disorder unless triggered by environmental factors like prolonged stress or lack of social support. The interaction between genetic vulnerability and environmental exposures is key to understanding why psychiatric disorders may appear or worsen in some individuals and not in others [42]. Thus, the heritability of psychiatric disorders should be viewed as a dynamic multifactorial process, where genetic factors provide susceptibility but environmental factors ultimately determine whether and when a disorder will manifest [43]. This understanding will help in shaping more comprehensive treatment and prevention strategies that consider both genetic predispositions and the environment’s influence.

### 2.2. Recent Findings

The role of gene–environment interactions is especially crucial in understanding the development of neuropsychiatric disorders. Recent research has demonstrated how environmental factors such as childhood trauma, stress, substance abuse, and infections interact with genetic predispositions to increase the risk of developing psychiatric disorders [44]. This gene–environment interaction is especially relevant in conditions like schizophrenia and bipolar disorder, where environmental stressors are known to trigger the onset or exacerbation of symptoms in genetically predisposed individuals [45]. Additionally, another study underscores the significance of polygenic factors in conditions like schizophrenia and autism. Advances in genome analysis, coupled with the formation of large patient cohorts, are enabling successful genetic investigations of polygenic brain disorders [21]. In order for the molecular insights, previously obscured in the genomes of affected individuals, to provide valuable information about disease mechanisms and contribute to the development of new treatments, the field of neurobiology must overcome several complex challenges. In this review, we explore the foundational principles behind this genetic investigation, delve into recent progress in understanding schizophrenia and autism, and examine the potential impact on future therapeutic strategies [46].

Additionally, recent findings on estrogen receptors (ER) in the female brain suggest that ER-mediated activation of kinases, including Akt and TrkB, plays a significant role in cell differentiation, survival, apoptosis, and neurodegenerative processes [47]. These pathways are involved in mental health conditions such as depression, schizophrenia, and neurodegenerative diseases, with both genetic and environmental factors influencing the expression of ERs. For instance, genetic variations in the ESR1 gene, which encodes the estrogen receptor α (ERα), have been linked to altered susceptibility to mood disorders [48]. Furthermore, environmental factors like hormonal fluctuations due to menopause, stress, or exposure to endocrine-disrupting chemicals can impact ER activity, potentially exacerbating mental health conditions [49]. Additionally, lifestyle factors such as diet and physical activity may influence estrogen metabolism and receptor expression, further affecting neuropsychiatric outcomes. For example, regular physical activity has been shown to increase ER activity, which may have protective effects against cognitive decline and psychiatric symptoms [50]. ER’s interaction with metabotropic glutamate receptors further suggests therapeutic potential for treating neuropsychiatric disorders, including Huntington’s disease, Alzheimer’s disease, Parkinson’s disease, schizophrenia, anxiety, and depression. ER agonists have shown promise in enhancing synaptic plasticity and improving memory and cognition, highlighting their potential for addressing neurological conditions related to synaptic dysfunction [51]. ER agonists have shown promise in enhancing synaptic plasticity and improving memory and cognition, highlighting their potential for addressing neurological conditions related to synaptic dysfunction [5].

Further, pharmacogenomics—the study of how an individual’s genetic profile influences their response to medications—has the potential to improve the efficacy of psychiatric drugs. Many commonly used antidepressants, antipsychotics, and mood stabilizers have varied effects across patients, often leading to trial-and-error approaches to finding the most effective medication. A previous study employed a pharmacometabolomic-informed pharmacogenomic strategy to identify biomarkers for citalopram/escitalopram treatment outcomes in major depressive disorder (MDD), revealing that plasma glycine levels were negatively associated with treatment response in remitters compared to non-remitters. This finding was further investigated through genotyping single nucleotide polymorphisms (SNPs) in genes involved in glycine synthesis and degradation in SSRI-treated MDD patients [52]. Moreover, integrating pharmacogenetic data into psychiatric practice offers a more tailored approach to treatment by addressing interindividual variability in drug response. For example, genetic tests that identify variations in cytochrome P450 enzymes, such as cytochrome P450 family 2 subfamily D member 6 and cytochrome P450 family 2 subfamily C member 19, can help clinicians predict how well a patient will metabolize certain medications, such as antidepressants and antipsychotics [53]. This can prevent drug toxicity or therapeutic failure and lead to more accurate dosing. Additionally, polymorphisms in the cytochrome P450 family 3 subfamily A member 4 gene have been linked to variability in the metabolism of antipsychotic drugs like olanzapine and clozapine. Similarly, the solute carrier organic anion transporter family member 1B1 gene, which affects drug transport, can influence the effectiveness of SSRIs and other psychotropic medications, ensuring that clinicians can make more informed decisions about drug choice and dosing based on a patient’s genetic profile [53].

The integration of these pharmacogenetic insights into clinical practice can be particularly useful in complex neuropsychiatric disorders, where the treatment response is often unpredictable and the side effects of medications can be severe. By using genetic testing, physicians could choose drugs that are most likely to be effective and safe for individual patients, improving patient adherence to treatment and overall mental health outcomes [54]. This approach represents a step forward in precision psychiatry, where treatments are customized not just to the individual’s symptoms but also to their genetic makeup, leading to more effective and personalized care.

By identifying genetic markers that influence drug metabolism and response, clinicians could more accurately predict which medications will be most effective for individual patients, reducing side effects and improving therapeutic outcomes. Furthermore, based on pharmacological studies, brain imaging analyses, and genetic research, tantalizing leads are now converging on the central role of several neurotransmitters—including dopamine, glutamate, and serotonin—that may interface with neurodevelopmental defects reflecting schizophrenia-related genetics [44]. Finally, regarding bipolar disorder, there is increasing evidence for the role of specific gene–environment interactions, as well as the mechanisms by which risk factors interact to lead to the onset of bipolar disorder [55]. We present an overview of the relationship between gene–environment interactions, pharmacogenomics, and personalized treatment in psychiatry. The most relevant diagnosis in regard to the hereditary aspect of mental disorders is in Table A1; we also mentioned this is the text.

Recent research has demonstrated how environmental factors such as childhood trauma, stress, substance abuse, and infections interact with genetic predispositions to increase the risk of developing psychiatric disorders. This gene–environment interaction is especially relevant in conditions like schizophrenia and bipolar disorder, where environmental stressors are known to trigger the onset or exacerbation of symptoms in genetically predisposed individuals. Additionally, another study underscores the significance of polygenic factors in conditions like schizophrenia and autism. Advances in genome analysis, coupled with the formation of large patient cohorts, are enabling successful genetic investigations of polygenic brain disorders [56]. In order for the molecular insights, previously obscured in the genomes of affected individuals, to provide valuable information about disease mechanisms and contribute to the development of new treatments, the field of neurobiology must overcome several complex challenges. In this review, we explore the foundational principles behind this genetic investigation, delve into recent progress in understanding schizophrenia and autism, and examine the potential impact on future therapeutic strategies.

#### 2.2.1. The Influence of Childhood Trauma on Neuropsychiatric Disorders

Childhood trauma, such as abuse or neglect, has been increasingly recognized as a significant environmental factor contributing to the development of neuropsychiatric disorders. A study by Muenke et al. (2020) found that early childhood trauma can alter brain structure and function, particularly in areas involved in emotional regulation, such as the prefrontal cortex and amygdala. These changes may interact with genetic predispositions, leading to a higher risk of developing conditions like post-traumatic stress disorder (PTSD), schizophrenia, or mood disorders. Moreover, this interaction between trauma and genetic risk factors has been shown to increase susceptibility to psychiatric symptoms later in life, especially when combined with additional environmental stressors [57].

Recent longitudinal studies, such as the work Williams et al. (2021), have shown that the interaction between genetic vulnerabilities and childhood trauma significantly increases the likelihood of developing major depressive disorder (MDD) and anxiety disorders. These findings suggest that early interventions aimed at mitigating the effects of trauma could potentially reduce the risk of psychiatric disorders by influencing both environmental and genetic pathways [58].

#### 2.2.2. Psychosis: Genetic and Environmental Interactions

Psychosis, characterized by distorted thinking, delusions, and hallucinations, is often a core symptom of severe mental illnesses like schizophrenia. A study Tamminga et al. (2022) revealed that psychosis can emerge from complex gene–environment interactions, with specific genetic variations heightening susceptibility to psychotic episodes triggered by environmental factors such as trauma, substance abuse, and stress. Furthermore, research by O’sullivan et al. (2009) highlighted the role of dopamine dysregulation in psychosis, demonstrating that genetic mutations affecting dopamine receptor signaling pathways can increase the likelihood of psychotic episodes when exposed to certain environmental stressors [56]. These findings emphasize the importance of understanding psychosis as a multifactorial condition, with both genetic and environmental contributions shaping its development and severity [59].

#### 2.2.3. Addiction: Genetic Markers and Environmental Triggers

Addiction, including substance use disorders, is another area where gene–environment interactions play a crucial role. Several studies have identified genetic markers that contribute to addiction susceptibility, particularly in relation to substances like alcohol, nicotine, and opioids. For instance, genetic variations in the ADH1B gene, which encodes an alcohol dehydrogenase enzyme, have been linked to alcohol dependence. Individuals carrying certain alleles of this gene have been found to have a reduced risk of alcohol dependence due to altered alcohol metabolism. Additionally, research by Weiss et al. (2020) has shown that genes involved in dopamine signaling, such as those related to the DRD2 receptor, are significant in the development of addiction, particularly in individuals exposed to stressful environments or early substance use.

Furthermore, a study by Kranzler et al. (2022) explored the gene–environment interaction in opioid addiction, finding that individuals with specific genetic variants in the OPRM1 gene, which encodes the mu-opioid receptor, are more likely to develop opioid dependence, especially when combined with early life trauma or social stressors. This underscores the complexity of addiction, which results from a dynamic interaction between genetic vulnerability and environmental influences, such as peer pressure, social isolation, and trauma.

#### 2.2.4. Autism Spectrum Disorder: Genetic Contributions and Environmental Modifiers

Autism spectrum disorder (ASD) is a neurodevelopmental disorder characterized by deficits in social communication and repetitive behaviors. Recent studies have demonstrated the significant role of genetics in the development of ASD. A large-scale study by Thompson et al. (2022) identified several novel genes associated with ASD, suggesting that mutations in these genes may disrupt neural circuits involved in social behavior and sensory processing. However, environmental factors, such as prenatal exposure to pollutants, maternal infections, and early childhood stress, also play a crucial role in modifying the expression of genetic risk factors. For example, a study by Wang et al. (2021) demonstrated that prenatal exposure to air pollution was associated with a higher risk of developing ASD in genetically predisposed children. These findings highlight the complexity of ASD and the need for an integrative approach that considers both genetic and environmental factors(Thompson et al., 2022).

#### 2.2.5. Depression and Bipolar Disorder: Genetic Vulnerabilities and Environmental Triggers

Depressive and bipolar disorders are characterized by disruptions in mood, cognition, and behavior, and both conditions have a well-established genetic basis. However, environmental factors, such as life stressors, social isolation, and substance abuse, can play a key role in triggering or exacerbating these disorders. A study by McGuffin et al. (2023) demonstrated that while genetic predispositions contribute to the risk of developing these disorders, stressful life events and interpersonal conflicts were significant triggers for the onset of depressive episodes. Similarly, research by found that the interaction between environmental stressors and genetic factors related to circadian rhythms may contribute to the development of bipolar disorder. This study suggests that therapies targeting circadian pathways could offer new treatments for bipolar disorder and mood disorders (McGuffin et al., 2023).

#### 2.2.6. The Role of Estrogen Receptors in Neuropsychiatric Disorders

Additionally, recent findings on estrogen receptors (ERs) in the female brain indicate that the ER-mediated activation of kinases, including Akt and TrkB, plays a role in cell differentiation, survival, apoptosis, and neurodegenerative processes, as well as mental health conditions such as depression, schizophrenia, and neurodegenerative diseases [60]. ER’s interaction with metabotropic glutamate receptors further suggests therapeutic potential for treating neuropsychiatric disorders, including Huntington’s disease, Alzheimer’s disease, Parkinson’s disease, schizophrenia, anxiety, and depression. ER agonists have shown promise in enhancing synaptic plasticity and improving memory and cognition, highlighting their potential for addressing neurological conditions related to synaptic dysfunction (Tamminga et al., 2022).

#### 2.2.7. Pharmacogenomics and Personalized Psychiatry

Pharmacogenomics—the study of how an individual’s genetic profile influences their response to medications—has the potential to improve the efficacy of psychiatric drugs. Many commonly used antidepressants, antipsychotics, and mood stabilizers have varied effects across patients, often leading to trial-and-error approaches to finding the most effective medication [61]. A previous study employed a pharmacometabolomics-informed pharmacogenomic strategy to identify biomarkers for citalopram/escitalopram treatment outcomes in major depressive disorder (MDD), revealing that plasma glycine levels were negatively associated with treatment response in remitters compared to non-remitters. This finding was further investigated through genotyping single nucleotide polymorphisms (SNPs) in genes involved in glycine synthesis and degradation in SSRI-treated MDD patients (Fabbri et al., 2021). By identifying genetic markers that influence drug metabolism and response, clinicians could more accurately predict which medications will be most effective for individual patients, reducing side effects and improving therapeutic outcomes (Weiss et al., 2020).

#### 2.2.8. Advancements in Polygenic Risk Scores for Schizophrenia and Autism

Recent advancements in polygenic risk scoring (PRS) have led to more accurate predictions of schizophrenia and autism spectrum disorder (ASD) susceptibility. According to the study by Allen et al. (2022), PRS can now identify individuals at elevated risk for developing these disorders with greater precision. This technique integrates genetic data across multiple risk loci, providing insights into how multiple genetic variations combine to influence the likelihood of developing complex neuropsychiatric conditions. Notably, polygenic scores are most effective when used in conjunction with environmental data, allowing researchers to better understand the combined impact of genes and the environment on mental health (Allen et al., 2022).

#### 2.2.9. Genetic Contributions to Bipolar Disorder and Future Therapies

Finally, regarding bipolar disorder, there is increasing evidence for the role of specific gene–environment interactions, as well as the mechanisms by which risk factors interact to lead to the onset of bipolar disorder (Fabbri et al., 2021). Recent studies have identified several candidate genes involved in circadian rhythm regulation, which may influence the onset and course of bipolar disorder. These findings suggest that therapies targeting these pathways could offer new approaches to the treatment of bipolar disorder and other mood-related disorders (McGuffin et al., 2023).

### 2.3. Implications for Therapeutic Development

The integration of precision medicine into clinical practice offers numerous benefits for the treatment of neuropsychiatric disorders. Identifying the genetic variants that influence drug metabolism could significantly enhance the efficacy of treatments like antidepressants and mood stabilizers, reducing side effects and improving patient outcomes. Moreover, precision medicine holds promise for the development of targeted therapies aimed at correcting specific molecular abnormalities in the brain. For instance, gene therapy and RNA-based approaches could offer curative treatments for conditions like schizophrenia and bipolar disorder, addressing the underlying genetic causes of these conditions, rather than merely alleviating symptoms [55]. In recent years, the use of genomic data to develop personalized treatments has extended beyond oncology, making significant strides in cardiovascular diseases and neuropsychiatry. In neuropsychiatric disorders, the integration of precision medicine holds the potential to drastically improve treatment outcomes. For example, genetic variations in the serotonin transporter gene have been linked to differential responses to selective serotonin reuptake inhibitors (SSRIs) in patients with major depressive disorder (MDD) [62]. By identifying these genetic markers, clinicians can tailor antidepressant therapy to individual patients, improving both efficacy and reducing adverse effects.

Moreover, the increasing application of pharmacogenomics is not only enhancing drug therapy but also paving the way for the development of novel targeted treatments in neuropsychiatry. A study on the glutamate receptor gene, which is involved in schizophrenia, suggests that targeted gene editing techniques may offer new opportunities to correct specific molecular defects, addressing the root cause of the disorder rather than just managing its symptoms Similarly, research into the dopamine receptor gene has shown promise in identifying potential drug targets that could help mitigate the cognitive deficits associated with disorders like schizophrenia. These advancements underscore the importance of genomic data in the development of personalized precision therapies in neuropsychiatry, enabling clinicians to move beyond one-size-fits-all approaches.

In summary, as genomic data continue to evolve, they promise to transform the landscape of neuropsychiatric treatment by not only improving the accuracy of drug prescriptions but also by leading to innovative therapies aimed at curing or reversing the underlying genetic causes of these conditions.

### 2.4. Integrating Genetic, Environmental, and Social Determinants in Understanding Psychiatric Disorders and Public Health Implications

In response to the growing complexity of psychiatric disorders, it is becoming increasingly clear that understanding mental health requires an integrated approach that incorporates both genetic and environmental/social determinants. Genetic research has contributed to the identification of various risk factors for psychiatric conditions, such as gene mutations and variations in neurotransmitter systems [63]. However, these findings alone do not fully account for the onset or progression of mental health disorders. Environmental and social factors significantly shape the way these genetic predispositions manifest. For example, factors such as childhood abuse, socioeconomic stress, and lack of social support can act as environmental triggers that increase susceptibility to conditions like depression, anxiety, schizophrenia, and bipolar disorder.

Integrating environmental and social determinants into psychiatric research offers a more comprehensive understanding of these disorders, recognizing that they arise from the interplay of biological vulnerabilities and external factors [64]. This perspective highlights the importance of adopting a public health approach to mental health care, one that emphasizes the prevention of mental health disorders through the reduction in environmental and social risk factors. Public health policies that address childhood trauma, poverty, social isolation, and lack of access to mental health care can effectively mitigate the impact of genetic risk factors, potentially reducing the overall burden of psychiatric disorders in society.

Moreover, public health interventions that target these social determinants can promote mental well-being at a population level. For example, community-based mental health programs, better social support systems, and increased access to mental health education are crucial for improving mental health outcomes. The integration of genetic, environmental, and social factors in understanding psychiatric disorders also has significant implications for personalized treatment strategies. By considering all aspects of an individual’s life, clinicians can provide more effective and targeted therapies, reducing trial-and-error approaches in medication and therapy selection.

## 3. Challenges and Controversies

### 3.1. Challenges in Research

While the promise of precision medicine in neuropsychiatry is exciting, several challenges remain. One of the primary obstacles is the complexity of psychiatric disorders themselves. Epidemiological studies indicate that individuals with one type of mental disorder have an increased risk of subsequently developing other types of mental disorders [64]. These conditions might be influenced by a myriad of genetic, environmental, and neurobiological factors, making it difficult to pinpoint specific biomarkers or genetic variants that could be used in routine clinical practice. Additionally, the application of precision medicine in psychiatry requires significant infrastructure, including advanced genetic testing, data analysis tools, and interdisciplinary collaboration between clinicians, researchers, and bioinformaticians. The detailed overview of the potential benefits and challenges of incorporating precision medicine into the treatment of neuropsychiatric disorders, particularly through understanding genetic variants, targeted therapies, and gene therapy approaches, is provided in Table A2.

#### Evaluating the Long-Term Impact of Precision Medicine on Both Healthcare Expenditures and Patient Quality of Life

In addition to the ethical concerns surrounding personalized treatment approaches, evaluating the feasibility of integrating genomics into clinical practice garnered increasing criticism. Personalized medicine, particularly in the neuropsychiatric field, holds the potential for more effective and tailored treatments. However, it often comes with substantial costs, including the expenses associated with genomic testing, high-priced specialized medications, and advanced diagnostic technologies. These costs can create barriers to widespread adoption, particularly in healthcare systems with limited budgets.

Critics of personalized treatment approaches argue that the increased costs associated with genomic testing and tailored therapies may not always result in significantly improved patient outcomes [65]. For example, while precision medicine has shown promise in oncology, where targeted therapies are more directly aligned with genetic markers, its application in neuropsychiatric disorders is still evolving. The financial burden of providing personalized treatments might not always justify the clinical benefits, especially when considering the variability in treatment response and the complexity of neuropsychiatric conditions.

To address these concerns, it is essential for future research to focus on assessing the cost-effectiveness of genomic-based treatments. Studies that evaluate the long-term impact of precision medicine on both healthcare expenditures and patient quality of life are necessary to inform policy decisions. If these treatments can be shown to deliver both better outcomes and cost savings over time, personalized neuropsychiatric care may become more feasible on a larger scale.

### 3.2. Controversies or Divergent Opinions

There are differing perspectives on how effectively precision medicine can be applied in psychiatry. Some argue that the complexity of psychiatric disorders, combined with the insufficient understanding of how genetic and environmental factors interact, makes precision medicine overly ambitious in the near term. Ethical concerns, such as privacy issues regarding genetic testing and the potential for discrimination based on genetic information, also remain contentious. In summary, there are four broad ethical issues in psychiatric genetics: (1) the use of genetic testing to predict future health outcomes, (3) the psychosocial consequences of genetic testing, (4) the effect of genetic testing on family members and communities, and (5) the ethics of the use of emerging genetic technologies [18]. While many researchers believe that precision medicine will eventually transform psychiatric care, others contend that the field is still too nascent to yield meaningful clinical applications in the immediate future. We present a detailed overview of the various perspectives and ethical issues surrounding precision medicine and psychiatric genetics in Table A3.

### 3.3. Addressing the Limitations of Current GWAS Studies in Psychiatry and the Potential for Overestimating Genetic Contributions

Genome-wide association studies (GWAS) have significantly advanced the field of psychiatric genetics by identifying genetic variants associated with disorders such as schizophrenia, bipolar disorder, and depression. However, it is important to address the limitations of these studies, as they may lead to an overestimation of the genetic contributions to psychiatric conditions. One of the most notable limitations is the relatively small effect sizes of the genetic variants identified. Despite the large sample sizes involved, each genetic variant typically contributes only modestly to an individual’s overall risk of developing a psychiatric disorder. This raises concerns that current GWAS findings may exaggerate the role of genetics in the etiology of these disorders, potentially overlooking other contributing factors.

Additionally, the focus of GWAS has largely been on common genetic variants, which, while frequent in the population, explain only a small portion of the heritability of psychiatric disorders. This is often referred to as the “missing heritability” problem, where the vast majority of genetic contributions to psychiatric conditions remain unidentified. This limitation suggests that GWAS alone may not provide a complete picture of the genetic architecture of these disorders.

Another challenge is the lack of diversity in GWAS populations, with many studies predominantly involving individuals of European ancestry. This limits the generalizability of findings to other populations and may skew the results, as genetic risk factors can differ across ethnic and geographic groups. To address these limitations, it is crucial to integrate alternative methodologies, such as exploring gene–environment interactions and epigenetic factors, which could provide a more nuanced understanding of the complex genetic underpinnings of psychiatric disorders.

In conclusion, while GWAS has been a valuable tool in psychiatric genetics, its limitations must be considered in order to avoid overestimating the role of genetics and to promote a more comprehensive approach to understanding psychiatric disorders.

### 3.4. Addressing Individual Variability in Treatment Response Through Precision Medicine

In psychiatric medicine, the heterogeneity of disorders such as schizophrenia, depression, and bipolar disorder presents a significant challenge in treatment. Precision medicine seeks to personalize treatment by incorporating genetic, environmental, and clinical data, thereby addressing the individual variability that often complicates psychiatric care. This personalized approach can improve treatment outcomes by identifying biomarkers that predict how patients will respond to specific medications and therapies.

The role of pharmacogenomics is pivotal in this process. By analyzing how an individual’s genetic makeup influences drug metabolism and receptor sensitivity, clinicians can select the most effective medications while minimizing the risk of adverse effects [66]. For instance, certain genetic variants may affect how a patient metabolizes antidepressants or antipsychotics, influencing both the efficacy and safety of these drugs. Tailoring treatment based on genetic information not only increases the likelihood of therapeutic success but also reduces the need for trial-and-error prescribing.

Beyond genetic factors, precision medicine also incorporates environmental and psychosocial determinants. These factors, including socioeconomic status, life stressors, and early childhood experiences, significantly impact treatment response. For example, patients with a history of trauma or chronic stress may require more integrated approaches that combine pharmacological treatment with psychotherapy or social support interventions.

Therefore, precision psychiatry is an evolving field that combines genetics, pharmacogenomics, and environmental factors to create personalized treatment plans that reflect the full complexity of psychiatric disorders. This holistic approach offers the potential for more effective interventions and better management of individual variability in treatment response.

## 4. Future Directions

### 4.1. Research Needs

The clinical applicability of precision medicine in neuropsychiatry is an evolving field that requires continued research to address key challenges. Several areas of future investigation are critical to advancing personalized care. One essential direction is the identification of novel biomarkers that can reliably predict treatment responses across diverse patient populations. This will require large-scale longitudinal studies integrating genetic, environmental, and neuroimaging data. Furthermore, improving methods for data integration, such as developing more accurate models for gene–environment interactions, will be crucial for enhancing treatment outcomes.

Another important area of research is the development of personalized treatment strategies that account for individual differences in genetic makeup, environmental exposures, and psychosocial factors. Such approaches will require collaboration between geneticists, psychiatrists, and social scientists to create holistic patient-centered treatment models. Moreover, the application of artificial intelligence in precision psychiatry could help to accelerate these efforts by analyzing large datasets to reveal complex patterns in treatment response and mental health outcomes.

To address the current gaps in understanding and overcome challenges, future research should focus on several key areas. Firstly, there is a need for larger and more diverse population studies to identify additional genetic variants and biomarkers linked to psychiatric disorders. These studies should also focus on better understanding the gene–environment interactions that contribute to the onset and progression of these conditions. Additionally, advancing technologies in neuroimaging and genomics could help bridge the gap between genetic findings and clinical outcomes. Finally, further progress is expected to be driven by the use of new and emerging technologies, such as whole-genome and long-read sequencing, applied to large and diverse samples. Significant advancements in biological understanding will also require concurrent progress in functional genomics and proteomics, particularly when applied to the brain across different developmental stages. To effectively identify disease mechanisms and define new subtypes, these efforts will need to be complemented by detailed phenotypic data [19].

### 4.2. Potential Therapeutic Advances

**Figure A1:**
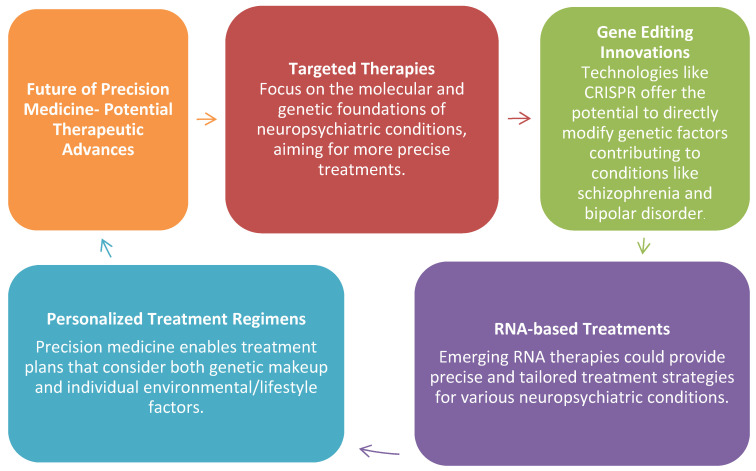
Potential Therapeutic Advance.

As research progresses, it is likely that targeted therapies will emerge, focusing on the molecular and genetic underpinnings of neuropsychiatric conditions. Innovations in gene editing, such as CRISPR, and RNA-based treatments could provide more precise therapeutic strategies for conditions like schizophrenia and bipolar disorder. Furthermore, precision medicine could pave the way for personalized treatment regimens that consider not only genetic makeup but also an individual’s unique environmental and lifestyle factors (Figure A1).

## 5. Conclusions

### 5.1. Summary

**Figure A2:**
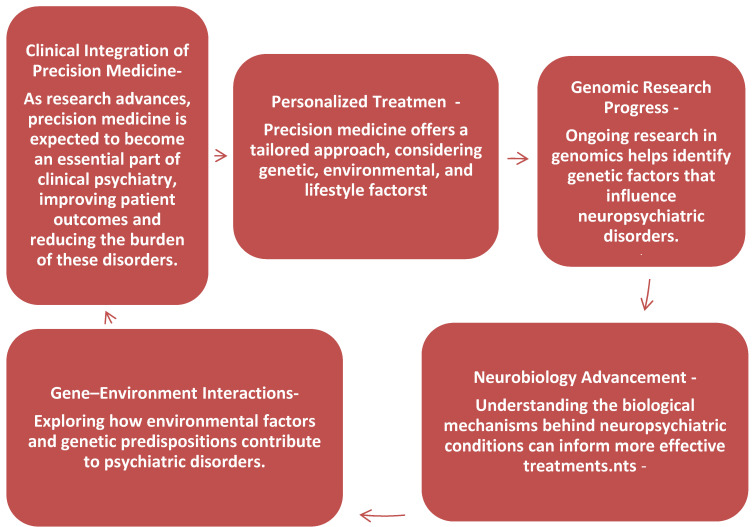
Clinical Integration of Precision Medicine.

In conclusion, precision medicine has the potential to revolutionize the treatment of neuropsychiatric disorders by providing a more personalized approach that accounts for genetic, environmental, and lifestyle factors. As research in genomics, neurobiology, and gene–environment interactions progresses, it is likely that precision medicine will become an integral part of clinical psychiatry, offering targeted treatments that improve patient outcomes and reduce the burden of these debilitating conditions (Figure A2 Clinical Integration of Precision Medicine).

### 5.2. Final Thoughts

While challenges remain, particularly in the areas of genetic complexity, data integration, and ethical considerations, the future of neuropsychiatry lies in the ability to unlock the pathophysiology of these disorders through precision medicine. With continued research and collaboration, the dream of personalized psychiatric care is within reach.

## Data Availability

Not applicable.

## Abbreviations

The following abbreviations are used in this manuscript:

CRISPR	Clustered regularly interspaced short palindromic repeats
GWAS	Genome-wide association studies
MDD	Major depressive disorder
RNA	Ribonucleic acid
SNP	Single nucleotide polymorphism
SSRIs	Selective serotonin reuptake inhibitors

## Appendix A

**Table A1:** The relationship between gene–environment interactions, pharmacogenomics, and personalized treatment in psychiatry.

Aspect of Gene–Environment Interactions, and Pharmacogenomic	Key Points	Informed Perspectives	Implications/Challenges
Gene–Environment Interactions	Crucial in understanding neuropsychiatric disorders. Environmental factors interact with genetic predispositions.	Environmental factors like childhood trauma, stress, substance abuse increase risk.	Important for conditions like schizophrenia and bipolar disorder, where environmental stressors trigger symptoms.
Environmental Stressors	Stress, trauma, substance abuse, and infections are key environmental factors in psychiatric disorders.	Stressors can trigger or exacerbate conditions in genetically predisposed individuals.	Stressors complicate diagnosis and treatment of psychiatric disorders, necessitating comprehensive care approaches.
Pharmacogenomics	The study of genetic profiles’ impact on medication response. Potential to improve drug efficacy.	Research on pharmacogenomics and its effect on medications like antidepressants, antipsychotics, and mood stabilizers.	Allows for personalized treatment, but still requires more research and clinical validation for broader application.
Pharmacometabolomics in MDD	Identifying biomarkers for treatment outcomes in major depressive disorder (MDD) using pharmacometabolomics.	Study on citalopram/escitalopram treatment outcomes in MDD, discovering plasma glycine levels’ association.	Could improve the precision of antidepressant treatments, but still in early research phases with ongoing studies needed.
Genetic Markers for Drug Response	Identifying genetic markers for better medication choices, reducing trial-and-error in prescribing drugs.	Genotyping SNPs involved in glycine synthesis and degradation in SSRI-treated MDD patients.	Potential to minimize side effects and improve treatment outcomes, but integration into clinical practice requires further development.
Pharmacometabolomics in Schizophrenia	Pharmacometabolomics can help identify biomarkers for treatment responses in schizophrenia, improving precision medicine.	Research exploring biomarkers specific to schizophrenia treatment responses, though still in exploratory phases.	Offers potential for more personalized treatment in schizophrenia, but further studies are required to validate findings.
Pharmacometabolomics in Bipolar Disorder	Focus on identifying biomarkers to predict responses to medications in bipolar disorder for improved therapeutic outcomes.	Research on how pharmacometabolomics can inform treatment plans for bipolar disorder, particularly mood stabilizers.	Can help tailor treatments, reducing the trial-and-error approach, but more research is necessary for practical application.

**Table A2:** The potential benefits and challenges of incorporating precision medicine into the treatment of neuropsychiatric disorders, particularly through understanding genetic variants, targeted therapies, and gene therapy approaches.

Topic	Key Points	Supporting Research/Concepts	Benefits	Challenges/Considerations
Precision Medicine in Clinical Practice	Integration of precision medicine can enhance treatment strategies for neuropsychiatric disorders.	Focus on genetic variants influencing drug metabolism.	Can improve treatment efficacy and reduce trial-and-error approaches.	Requires significant research and clinical validation; ethical concerns about genetic information use.
Genetic Variants and Drug Metabolism	Identifying genetic variants that affect how patients metabolize medications can lead to personalized treatments.	Variants in drug-metabolizing enzymes (e.g., CYP450 enzymes) affect the metabolism of antidepressants, antipsychotics, etc.	Reduces side effects and improves drug efficacy, optimizing treatment for individual patients.	Complex genetic profiles make it difficult to predict individual responses accurately without extensive research.
Antidepressants and Mood Stabilizers	Pharmacogenomics can guide medication choices for antidepressants and mood stabilizers, enhancing outcomes	Studies on genetic influences on drug responses for conditions like depression and bipolar disorder.	More precise targeting of medications can minimize side effects and increase effectiveness for patients.	Medications may still require adjustments due to individual variations not captured by current genetic tests.
Targeted Therapies	Development of therapies that target specific molecular abnormalities in the brain, aiming for more effective treatments.	Emerging research on gene therapy and RNA-based approaches for psychiatric conditions.	Address underlying causes of conditions like schizophrenia and bipolar disorder, offering curative potential.	High cost, ethical concerns, and long-term safety implications need to be considered before widespread use.
Gene Therapy in Neuropsychiatric Disorders	Gene therapy aims to correct genetic mutations or molecular abnormalities that contribute to conditions.	Promising research in gene editing technologies (e.g., CRISPR) for neuropsychiatric conditions like schizophrenia.	Potential for long-term cures and reduction in symptoms through direct genetic intervention.	Risk of unintended genetic consequences, lack of comprehensive understanding of the long-term effects on brain function.
RNA-based Approaches	RNA-based therapies could be developed to modify gene expression or correct genetic defects.	Research into RNA interference (RNAi) and antisense oligonucleotides as potential treatments for neuropsychiatric conditions.	Could offer a more targeted and non-invasive approach to correcting genetic defects in neuropsychiatric disorders.	Challenges in delivery mechanisms, ensuring the specificity of treatment, and preventing off-target effects.
Addressing Underlying Genetic Causes	A shift from symptom management to addressing the root causes of neuropsychiatric disorders.	Genetic studies identifying molecular mechanisms behind conditions like schizophrenia, bipolar disorder, etc.	Provides potential for curative treatments rather than merely alleviating symptoms.	Still in experimental phases; the complexity of gene–environment interactions complicates direct cures.

**Table A3:** The various perspectives and ethical issues surrounding precision medicine and psychiatric genetics.

Issues of Precision Medicine	Perspective	Supporting Arguments	Challenges/Concerns
Assessment of Potentials of Precision Medicine in Psychiatry	Overly ambitious in the near term	Complexity of psychiatric disorders; insufficient understanding of genetic-environment interactions	Clinical applications are still too nascent for meaningful impact
Genetic Testing to Predict Health Outcomes in Psychiatry	Risk of being prematurely applied	Can provide valuable predictions for future health outcomes	Ethical concerns, such as privacy and discrimination based on genetic information
Psychosocial Consequences of Genetic Testing Findings	Potential negative impact on individuals	Genetic testing could lead to changes in identity and personal responsibility	Psychological distress, stigma, and anxiety about genetic predispositions
Psychosocial Consequences of Genetic Testing Findings—Effects on Family Members and Communities	Unpredictable effects on others	Genetic information can influence family dynamics and societal relationships	Potential for discrimination, familial tensions, and societal stigma
Ethics of Emerging Genetic Technologies	Ethical dilemmas of unregulated use	Emerging technologies could improve psychiatric treatment	Unclear ethical guidelines, risks of misuse, and unintended consequences
